# Intravenous Administration of Bone Marrow-Derived Mesenchymal Stem Cell, but not Adipose Tissue-Derived Stem Cell, Ameliorated the Neonatal Hypoxic-Ischemic Brain Injury by Changing Cerebral Inflammatory State in Rat

**DOI:** 10.3389/fneur.2018.00757

**Published:** 2018-09-11

**Authors:** Yuichiro Sugiyama, Yoshiaki Sato, Yuma Kitase, Toshihiko Suzuki, Taiki Kondo, Alkisti Mikrogeorgiou, Asuka Horinouchi, Shoichi Maruyama, Yoshie Shimoyama, Masahiro Tsuji, Satoshi Suzuki, Tokunori Yamamoto, Masahiro Hayakawa

**Affiliations:** ^1^Division of Neonatology, Center for Maternal-Neonatal Care, Nagoya University Hospital, Nagoya, Japan; ^2^Department of Nephrology, Nagoya University Graduate School of Medicine, Nagoya, Japan; ^3^Pathology and Clinical Laboratories, Nagoya University Hospital, Nagoya, Japan; ^4^Department of Regenerative Medicine and Tissue Engineering, National Cerebral and Cardiovascular Center, Osaka, Japan; ^5^Center for Advanced Medicine and Clinical Research, Nagoya University Graduate School of Medicine, Nagoya, Japan; ^6^Department of Urology, Nagoya University Graduate School of Medicine, Nagoya, Japan; ^7^Laboratory for Clinical Application of Adipose-Derived Regenerative Cells, Nagoya University Graduate School of Medicine, Nagoya, Japan

**Keywords:** neonatal encephalopathy, regenerative medicine, cytotherapy, M1 microglia, serum chemokine, multiplex, cell distribution

## Abstract

Perinatal hypoxic-ischemic (HI) brain injury occurs in 1 in 1,000 live births and remains the main cause of neurological disability and death in term infants. Cytotherapy has recently emerged as a novel treatment for tissue injury. In particular, mesenchymal stem cells (MSCs) are thought to have therapeutic potential, but little is known about the differences according to their origin. In the current study, we investigated the therapeutic effects and safety of intravenous injection of allogeneic bone marrow-derived MSCs (BM-MSCs) and adipose-derived stem cells (ADSCs) in a rat model of HI brain injury. HI models were generated by ligating the left carotid artery of postnatal day 7 Wistar/ST rats and exposing them to 8% hypoxia for 60 min. Bone marrow and adipose tissue were harvested from adult green fluorescent protein transgenic Wistar rats, and cells were isolated and cultured to develop BM-MSCs and ADSCs. At passaging stages 2–3, 1 × 10^5^ cells were intravenously injected into the external right jugular vein of the HI rats at 4 or 24 h after hypoxia. Brain damage was evaluated by counting the number of cells positive for active caspase-3 in the entire dentate gyrus. Microglial isotypes and serum cytokines/chemokines were also evaluated. Distribution of each cell type after intravenous injection was investigated pathologically and bio-optically by *ex vivo* imaging (IVIS®) with a fluorescent lipophilic tracer DiR. The mortality rate was higher in the ADSC group compared to the BM-MSC group, in pups injected with cells 4 h after hypoxia. The number of active caspase-3-positive cells significantly decreased in the BM-MSC group, and the percentage of M1 microglia (a proinflammatory isotype) was also lower in the BM-MSC vs control group in the penumbra of the cortex. Moreover, BM-MSC administration increased anti-inflammatory cytokine and growth factor levels, while ADSCs did not. Each injected cell type was mainly distributed in the lungs and liver, but ADSCs remained in the lungs longer. Pathologically, pulmonary embolisms and diffuse alveolar hemorrhages were seen in the ADSC group. These results indicated that injection of allogeneic BM-MSCs ameliorated neonatal HI brain injury, whereas ADSCs induced severe lung hemorrhage and higher mortality.

## Introduction

Perinatal hypoxic-ischemic (HI) brain injury occurs in 1 in 1,000 live births and remains a main cause of neurological disabilities and death in term infants (1, 2). Therapeutic hypothermia is the only established treatment option; however, its effect is limited (3–5). Meanwhile, cytotherapy has been emerging as a novel therapy for HI. Recently, we demonstrated the beneficial effect of umbilical cord blood mononuclear cells in rat neonatal HI and mouse stroke models (6–8), and autologous umbilical cord blood cells therapy is now at the clinical trials stage(ClinicalTrials.gov: NCT02256618) (9). However, in situations where asphyxiated babies are born, there is a chance of failure of cord blood collection owing to the clinical staff being busy treating the mother and resuscitating the infant. Allogeneic cell transplantation should be considered as a treatment for such asphyxiated infants.

In animal models, neural stem cells have been shown to have a powerful effect on regeneration of damaged brain regions (10, 11). However, ethical issues discourage their clinical use because such treatments require a fetal brain to obtain the neural stem cells. As a result, other cell types, such as bone marrow-derived mesenchymal stem cells (BM-MSC) and adipose tissue-derived stem cells (ADSC), have been investigated and employed as alternatives. MSCs are thought to be a practical cell source, and many studies have demonstrated that they attenuate tissue damage in various inflammation and/or ischemic models (12–17). The beneficial effect of MSC transplantation is related to their potency to differentiate into multiple lineages (18). Their administration has been shown to improve the tissue environment via endocrine/paracrine effects (19, 20) and have immunosuppressive effects (21, 22).

In particular, ADSCs have some advantages over BM-MSC as adipose tissue is easy to collect under local anesthesia (liposuction), can be collected repeatedly (23), and greater numbers can be collected at one time. Moreover, ADSCs proliferate faster than BM-MSCs (24). Despite the numerous reports about the efficacy of ADSCs (25, 26) and their use in clinical trials (27), there is still no report on their use for neonatal HI. On the other hand, there is some debate about the safety of intravenous administration of ADSCs. Recent reports warn of embolism after intravenous ADSC administration (28), and increased coagulation activity after transplantation (29, 30) is thought to be an underlying cause. In addition, cellular distribution after intravenous administration of ADSCs has not been well-investigated, especially in the subacute/chronic phase, raising safety concerns, such as risk of pulmonary embolism. In the current study, we investigated the safety and efficacy of intravenous administration of ADSCs and BM-MSCs in a rat model of neonatal HI brain injury.

## Materials and methods

### Animals

This study was carried out in accordance with the Regulations on Animal Experiments in Nagoya University. The protocol was approved by the Institutional Review Board of Animal Experimentation of Nagoya University School of Medicine (Nagoya, Japan; Protocol No.: 24337-2012, 25170-2013, 26128-2014). Wister/ST rats (SLC Inc., Shizuoka, Japan) were used for the HI model. MSCs were harvested from green fluorescent protein (GFP)-Transgenic (Tg) Wistar rats which were supplied by the National BioResource Project-Rat, Kyoto University (Kyoto, Japan). All rats were maintained under a 12 h light/12 h dark cycle (lights on from 9:00 AM to 9:00 PM) with *ad libitum* access to food and water. Every effort was made to reduce animal suffering.

### Hypoxic-ischemic brain injury animal model

HI rat models were made according to the method of Rice et al. (31) with minor modification as described in our previous reports (7, 32). On postnatal day 7 (P7), Wistar/ST male and female rat pups were anesthetized with isoflurane and their left common carotid artery was double-ligated with 5-0 surgical silk and cut between the ligatures. The anesthesia time never exceeded 10 min for each pup. After a 1 h rest with dam, they were exposed to 8% hypoxia at 37 C in an incubator for 60 min.

### Cell preparation

For preparation of BM-MSCs, 3- to 5-week-old female GFP-Tag Wistar/ST rats were anesthetized with isoflurane and their femurs and tibias were removed aseptically. Then, heparinized saline was used to flush the marrow shafts using a 23-G needle, and the bone marrow suspension was harvested. After washing with 0.1 mM EDTA-saline, cells were resuspended in 5 mL of Minimal Essential Medium (MEM) alpha (Invitrogen, Carlsbad, CA, USA) with 2% albumin (Japan Blood Products, Tokyo, Japan). Mononuclear cells were isolated with Ficoll®-Paque PLUS (GE Healthcare Life Sciences, Uppsala, Sweden). To culture BM-MSCs, mononuclear cells were suspended in 5 mL MEM alpha with 20% FBS (Thermo Fisher Scientific, Waltham, MA, USA), and plated at 4–6 × 10^6^ cells per 25-cm^2^ flask and incubated at 37°C in a humidified atmosphere with 5% CO_2_ for 1–2 weeks until the first passage. We selected these plastic-adherent cells as BM-MSCs. BM-MSCs were used for injection after the second or third passage.

ADSCs were also prepared from 3- to 5-week-old female GFP-Tag Wistar/ST Rats. Rats were gently killed by CO_2_ asphyxiation, and adipose tissues were obtained from the fatty layer of the subcutaneous tissue. Generally, 2–4 g of adipose tissue was obtained from each rat. Adipose tissue was well-minced in MEM alpha (Gibco®) and digested with 1 mg/mL collagenase type II solution (Invitrogen) with stirring for 1 h at 37°C. The digested tissue was filtered using a 100-μm cell strainer. Then stromal vascular fraction was precipitated by centrifugation at 1,200 rpm for 5 min at room temperature then washed twice with MEM alpha containing FBS and antibiotics. Stromal vascular fraction cells were seeded (2 × 10^6^ cells) in 225-cm^2^ T-flasks and cultured in Dulbecco's MEM (Gibco®) containing 20% FBS at 37°C in a humidified atmosphere with 5% CO_2_ and 95% air. Four to Five days later, unattached cells were removed, and the medium changed to Dulbecco's MEM containing 3% FBS. Cells were collected from culture flasks at 90% confluence using 0.05% trypsin-EDTA (Wako, Osaka, Japan) and reseeded at 1,000 cells/cm^2^ to ensure optimal proliferation. ADSCs were used for injection after the second or third passage.

### Intravenous injection of cells

Rats were set on an electric warmer plate to maintain proper body temperature and anesthetized with inhaled isoflurane. Then, the skin was cut to expose the right external jugular vein. ADSCs or BM-MSCs were injected slowly into the vein using a 35-G needle; cells were suspended in 0.1 mL phosphate-buffered saline (PBS) and kept on ice until being rewarmed to room temperature just before injection. To evaluate the treatment effect, each cell or vehicle was administered at 24 h after HI. To assess the mortality and pathological findings, cells and vehicle were given at 4 or 24 h after HI.

### Cell labeling with DIR

Injected cells were labeled with the fluorescent tracer 1, 1-dioctadecyl-3,3,3,3-tetramethyl indotricarbocyanine iodide (DiR; Caliper Life Sciences, Hopkinton, MA, USA) following to the manufacturer's protocol. Briefly, cells were incubated with DiR for 30 min at 37°C, centrifuged for 5 min at 1,500 rpm at room temperature, and then rinsed twice with PBS. In all the cases, DiR-labeled cells were suspended in PBS, and 1 × 10^5^ cells were injected within 2 h after labeling.

### *Ex vivo* imaging and analysis

To reduce fluorescent noise, all rats used for *ex vivo* imaging were fed an alfalfa-free diet (D10001, Research Diets Inc., New Brunswick, NJ, USA). DiR-labeled BM-MSCs (*n* = 18) or ADSCs (*n* = 18) were intravenously injected into neonatal HI rats 24 h after the hypoxic insult. Of these, three rats from each cell type-treatment group were sacrificed at 1 h, 1 d, 3 d, 7 d, 14 d, and 28 d after injection to collect brain, lungs, heart, liver, spleen, gut, kidney, and bladder for *ex vivo* imaging. The collected organs were imaged using IVIS® Spectrum (Caliper Life Sciences). Filter conditions and illuminations settings for DiR imaging were set an excitation/emission of 710/760 nm, high lamp level, medium binning, filter 1, and 1.0 sec exposure time. Grayscale and fluorescent images of each organ were analyzed using Living Image software version 4.3 (Xenogen). Regions of interest of each organ were automatically drawn over the signals on images and, if necessary, they were manually corrected according to the grayscale image. Quantification was made according to the method of Cho et al. (33) with modification. The distribution of each DiR-labeled cell in each organ was quantified as the average radiant efficiency (total photons/s/cm^2^/steradian) in the irradiance range (μW/cm^2^): (photons/s/cm^2^/steradian)/(μW/cm^2^). To reduce variability in measurements, the ratio of the average radiant efficiency of the organs to the background was calculated. The minimum detectable fluorescence required 1 × 10^3^ cells as in a previous report (34).

### Immunohistochemistry

Immunostaining of brain sections with anti-active caspase-3 was performed as previously described (35) with minor modifications. Briefly, rats were anesthetized with pentobarbital (Kyoritsu Seiyaku Co., Tokyo, Japan) and intracardially perfusion-fixed with 0.9% NaCl, followed by 4% paraformaldehyde in PBS. Then, brains were immersion-fixed in 4% paraformaldehyde in PBS at 4°C for 24 h, dehydrated with a graded series of ethanol and xylene, embedded in paraffin, and cut into 5-μm-thick coronal sections. After deparaffinization and rehydration, antigen retrieval was performed by heating sections for 10 min in 10-mM citrate buffer (pH 6.0). Then, sections were blocked in PBS containing 0.1% Triton and 4% donkey serum and incubated overnight at 4°C with rabbit anti-active caspase-3 (1:200; BD Pharmingen, Franklin Lakes, NJ, USA). Sections were subsequently incubated with a donkey anti-rabbit biotinylated secondary antibody (Vector Laboratories, Burlingame, CA, USA) for 1 h at room temperature. Endogenous peroxidase activity was blocked with 3% H_2_O_2_ in PBS for 10 min and then an avidin-biotin-peroxidase complex (Vectastain ABC Elite kit; Vector Laboratories), followed by peroxidase detection for 10 min (0.12 mg/mL 3,3'-diaminobenzidine, 0.01% H_2_O_2_, and 0.04% NiCl_2_). For immunostaining of cells injected into the lungs, rats were euthanized by decapitation, and the lungs were removed and fixed with 4% paraformaldehyde in PBS. Subsequent immunostaining procedures were performed in the same way as for brain sections, except lung sections were incubated with a rabbit anti-GFP (1:200; MBL) primary antibody. To evaluate microglia, two brain sections per pup at the hippocampal and basal ganglia level were used. After antigen retrieval and blocking of nonspecific binding, sections were incubated with anti-ionized calcium-binding adapter molecule (Iba) 1 (1:100; Wako) and anti-inducible nitric oxide synthase (iNOS; 1:40; Abcam, Cambridge, MA, USA) primary antibodies at 4°C overnight. Sections were subsequently incubated with Alexa-548 and Alexa-488 for 1 h at room temperature, and mounted with ProLong Gold Antifade reagent containing DAPI (Thermo Fisher Scientific Inc.).

### Cell counting

For counting of anti-active caspase-3-positive cells, every 50th section at the hippocampus level (typically 5 sections) were stained using an anti-active caspase-3 primary antibody (1:1000). The hippocampal CA3 and dentate gyrus were outlined under low magnification (40×), and active caspase-3-positive cells in these areas were counted under high magnification (200×) using Stereo Investigator version 10 stereology software (MicroBrightField Europe EK, Magdeburg, Germany). The total number of cells was calculated using the following formula: *N* = Σ*A* × *P*, where *N* = the total number of cells, Σ*A* = the sum of the counted number of cells, and *P* = the inverse of the sampling fraction.

For evaluation of microglial M1 polarization, Iba1- (pan-microglia marker) and iNOS- (M1 microglia marker) positive cells were counted. M1 polarity was calculated by the percentage of iNOS/Iba1 double-positive cells in Iba1-positive cells (36). All positive cells were counted within a 200-μm^2^ area in the hippocampus (CA3), basal ganglia, and upper/lower side of the penumbra of the cortex in the two sections.

### Serum cytokine, chemokine, and growth factor analyses

Blood samples were collected from the heart at sacrifice for immunohistochemical evaluation. To obtain serum samples, blood samples were immediately centrifuged and kept on ice until freezing. Serum samples were analyzed by MILLIPLEX® Multiplex Assays using Luminex® with a rat cytokine/chemokine panel (Merck Millipore, Billerica, MA, USA) according to the manufacture's protocol. The MILLIPLEX® plate was read with Luminex MagPix technology. Data was analyzed using xPONENT® software (Luminex, Austin, TX, USA).

### Pathology

ADSCs or BM-MSCs (1 × 10^4^, 1 × 10^5^ or 1 × 10^6^ cells) were injected intravenously 4 h after HI insult. Organs of interest were excised 15 min after injection without perfusion then fixed in paraformaldehyde, embedded in paraffin, cut into 10-mm sections, and stained with hematoxylin-eosin. Pathological findings were determined by our pathologist (Y Shimoyama).

### Statistical analyses

The sample size was decided to be 5 to 10 in each group based on our previous studies (7, 32). Statistical analyses were performed using JMP11.0 software (SAS Institute, Cary, NC, USA). Mortality after injection was compared using a Fisher's exact test. For analyses of immunohistochemistry and serum cytokine/chemokine and growth factor levels, one-way analysis of variance was used, followed by a Dunnett's *post-hoc* test. Two-group analyses of organ fluorescence by *ex vivo* imaging were compared using a Student's *t*-test. All data are expressed as the mean ± standard error of the mean. A *P*-value of less than 0.05 was considered statistically significant.

## Results

### Mortality after administration of ADSC or BM-MSC

To assess the safety of each cytotherapy, ADSCs or BM-MSCs (1 × 10^5^ cells/0.1 mL PBS) or vehicle (0.1 mL PBS) were given 4 or 24 h after HI insult. The mortality rate within 24 h after administration was significantly higher in the ADSC group (64%) than the BM-MSC group (6%) when cells were given 4 h after hypoxia exposure (Table 1). However, there was no significant difference among ADSC, BM-MSC, and vehicle groups when cells were given 24 h after hypoxia exposure.

**Table 1:** Mortality within 24 h after administration of MSCs.

**Time of cell administration after HI (hour)**	**4**			**24**		
**Agent**	**Vehicle**	**ADSC**	**BM-MSC**	**Vehicle**	**ASC**	**BM-MSC**
given cell count	–	1 × 10^5^	1 × 10^5^	–	1 × 10^5^	1 × 10^5^
Number of the rat (dead within 24 h/total)	0/6	9/14	1/16	0/31	7/85	2/42
Mortality within 24 h	0%	64%	6%	0%	8%	5%
Fisher's exact test vs. Vehicle	–	*p* < 0.01	n.s.	–	n.s.	n.s.

### Impact of ADSC and BM-MSC administration on apoptosis after HI

**Figure 1:**
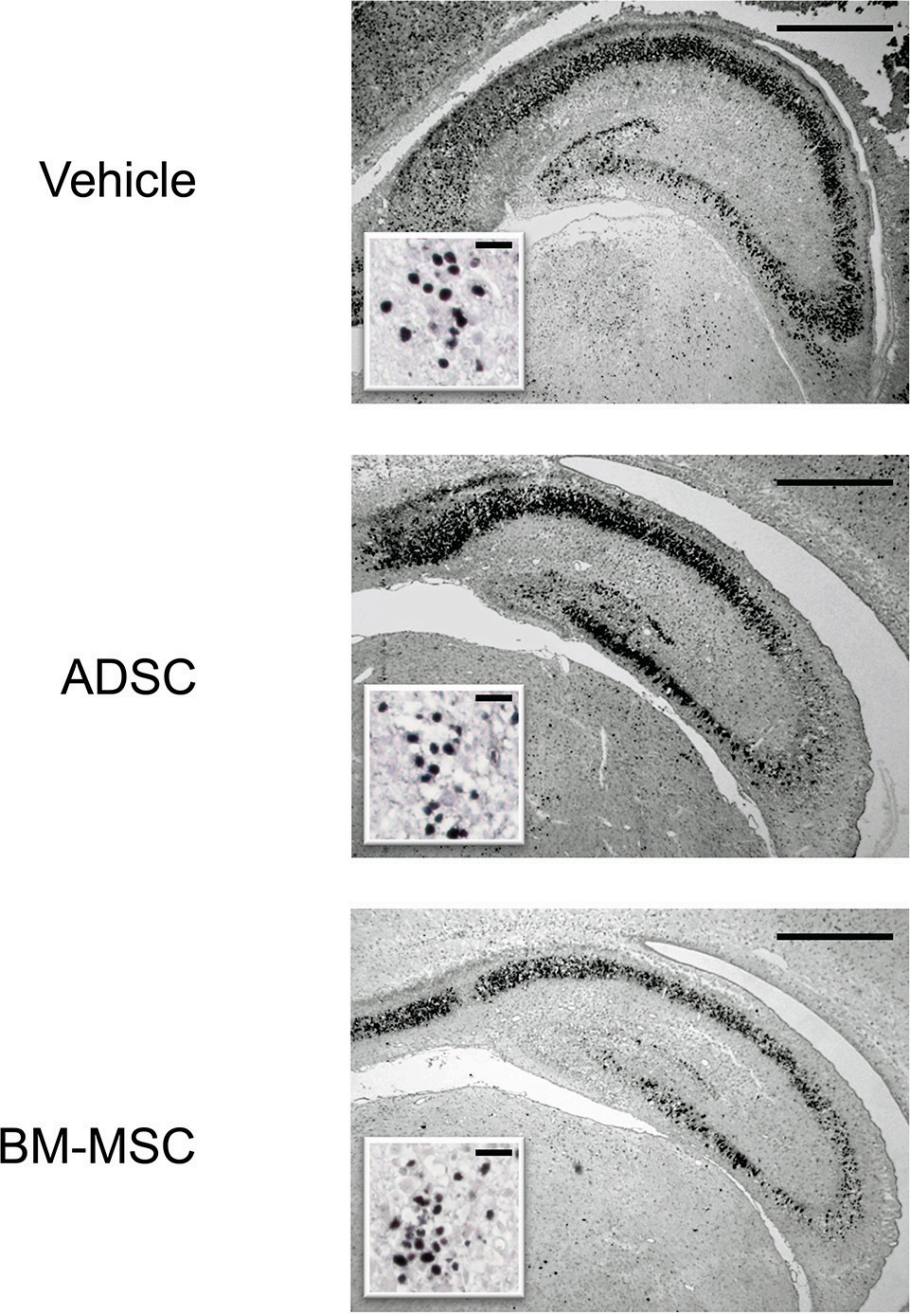
Photomicrographs of anti-active caspase-3 staining of the hippocampus. Representative photomicrographs of the hippocampus 48 h after HI insult. Sections are from rats injected with vehicle (PBS), ADSCs, or BM-MSCs; bar = 500 μm. Insets show higher magnification; bar = 50 μm.

**Figure 2:**
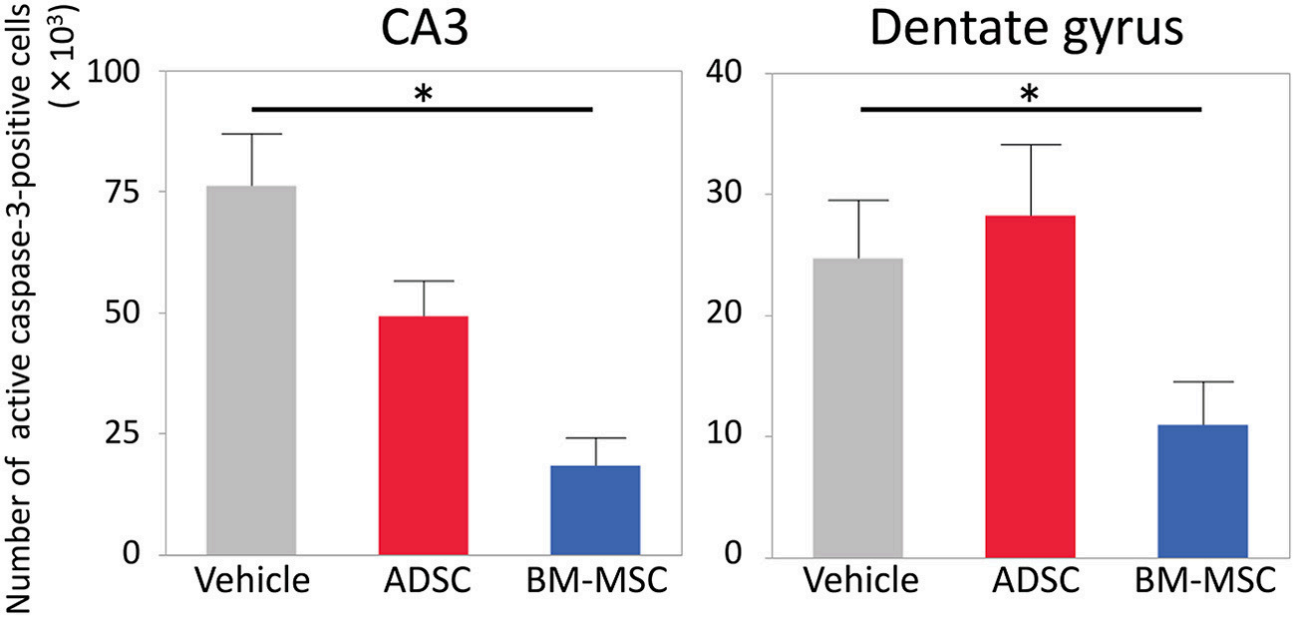
Number of active caspase-3-positive cells in the CA3 and dentate gyrus of the affected side. Impact of vehicle (PBS) (*n* = 7), ADSCs (*n* = 7), and BM-MSCs (*n* = 8) on apoptosis 24 h after injection. The number of active caspase-3-positive cells was significantly decreased in BM-MSC rats but not ADSC rats compared with vehicle. *P < 0.05.

Twenty-four hours after HI, P7 rats were injected with 1 × 10^5^ ADSCs (*n* = 8), BM-MSCs (*n* = 7), or vehicle (*n* = 8). The rats were sacrificed 24 h after injection, and the numbers of active caspase-3-positive cells in the CA3 area and entire dentate gyrus were counted. Photomicrographs of representative hippocampal sections are shown in Figure 1. The number of active caspase-3-positive cells significantly decreased in CA3 area and dentate gyrus by 76% (*P* < 0.05) and 59% (*P* < 0.05), respectively, in the BM-MSC group but not in the ADSC group (Figure 2).

### Microglial M1 polarization after administration of ADSCS and BM-MSCS

**Figure 3:**
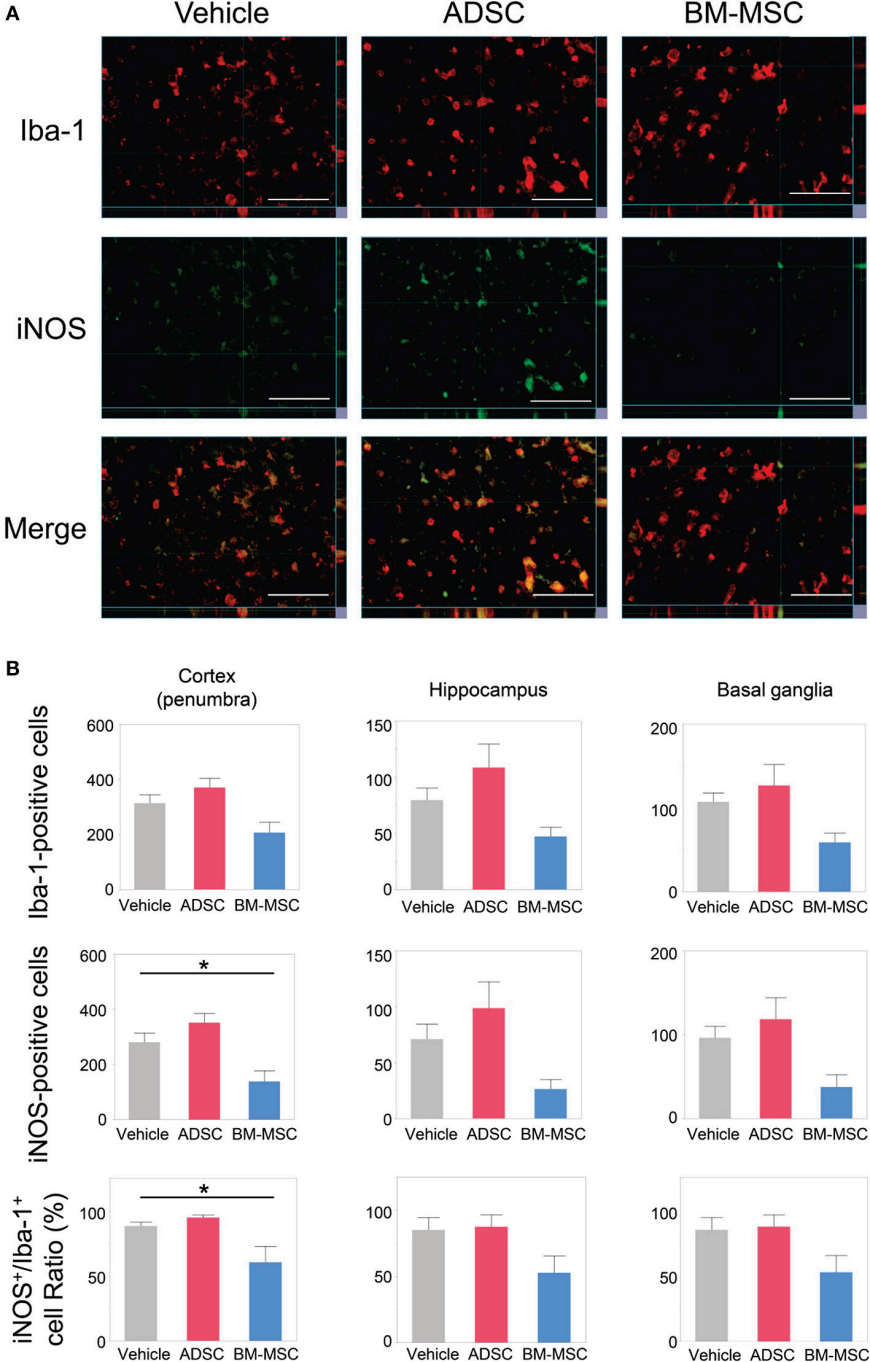
Impact of BM-MSCs and ADSCs on microglial M1 polarization in the cortex, hippocampus, and basal ganglia. **(A)** Representative photomicrographs of the affected side cortex stained with anti-Iba-1 (pan-microglia marker) and anti-iNOS (M1 phenotype marker) 24 h after injection. Bar = 50 μm. **(B)** Number of Iba-1/iNOS positive cells, and the ratio of iNOS to Iba-1 in the penumbra of the cortex, hippocampus, and basal ganglia. In the penumbra of the cortex, iNOS-positive cell and the iNOS/Iba-1 ratio were significantly decreased in BM-MSC rats (*n* = 8), but not in ADSC rats (*n* = 7), compared to those in the vehicle group (*n* = 7). These parameters in hippocampus basal ganglia, and the number of Iba-1 in penumbra of the cortex and hippocampus showed the same trend. *P < 0.05.

To evaluate the impact of ADSCs and BM-MSCs on microglial M1 polarization, we double-stained brain sections with anti-Iba1 and anti-iNOS antibodies. Then, the number of Iba1/iNOS double-positive cells and Iba1-positive/iNOS-negative cells was counted. Representative photomicrographs are shown in Figure 3A. The number of Iba1-positive cells in the BM-MSC group tended to decrease in the penumbra of the cortex, hippocampus, and basal ganglia but was not statistically significant (Figure 3B); this was not observed in the ADSC group.

In the BM-MSC group, the number of Iba1/iNOS double-positive cells and the ratio of Iba1/iNOS double-positive to Iba1-positive cells were found to be significantly decreased in the penumbra of the cortex compared to the vehicle group (both, *P* < 0.05; Figures 3A,B); no significant change was found for the ADSC group. The same trend was seen in the hippocampus and basal ganglia. This indicates that intravenous injection of BM-MSCs, but not ADSCs, decreased the polarity of M1 microglia.

### Impact of ADSC and BM-MSC administration on serum cytokines/chemokines

**Figure 4:**
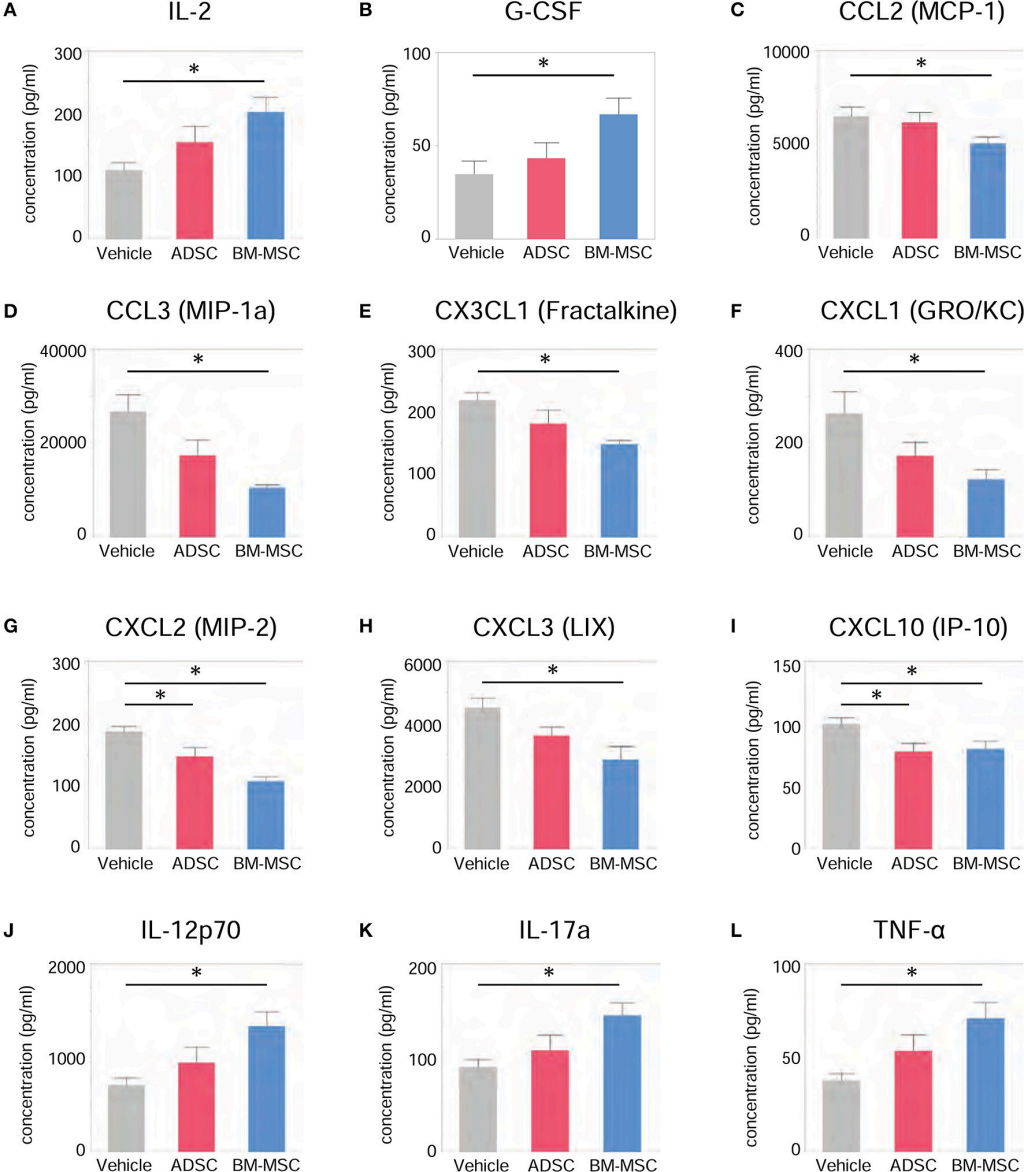
Impact of BM-MSCs and ADSCs on serum cytokines/chemokines. Various serum cytokines/chemokines were measured by multiplex assay 24 h after injection. Serum levels of various cytokines/chemokines were significantly altered in the BM-MSC group but not the ADSC group. BM-MSCs, but not ADSCs, increased anti-inflammatory cytokine IL-2 **(A)** and granulocyte colony stimulating factor **(B)** levels. BM-MSCs also decreased chemotactic chemokines CCL2 **(C)**, CCL3 **(D)**, CX3CL1 **(E)**, CXCL1 **(F)**, CXCL2 **(G)**, CXCL3 **(H)**, and CXCL10 **(I)**. On the other hand, levels of inflammatory cytokines IL-12p70 **(J)**, IL-17a **(K)**, and tumor necrosis factor **(L)** were increased by BM-MSC administration, but not ADSC. *P < 0.05.

To evaluate the serological effect of BM-MSCs or ADSCs, serum cytokine/chemokine levels 24 h after injecting ADSCs or BM-MSCs were analyzed by multiplex assay (MILLIPLEX®). All cytokine/chemokine levels measured are listed in Table 2. BM-MSC administration significantly increased anti-inflammatory cytokine interleukin (IL)-2 level compared to vehicle (Figure 4A). And anti-inflammatory cytokine IL-4 also tended to be increased, but not significantly (*p* = 0.068). Granulocyte colony stimulating factor (G-CSF) levels were also significantly elevated in the BM-MSC group compared with control (Figure 4B). BM-MSCs also significantly reduced the levels of several chemotactic chemokines, including CCL2 (monocyte chemoattractant protein-1), CCL3 (macrophage inflammatory protein-1a), CX3CL1 (Fractalkine), CXCL1 (human growth-regulated oncogene/keratinocyte chemoattractant), CXCL2 (monocyte inflammatory protein-2), CXCL3 (lipopolysaccharide-induced CXC chemokine), and CXCL10 (interferon-γ-induced protein-10) [Figures 4C–I]. In contrast, BM-MSCs significantly increased three inflammatory cytokines, IL-12p70, IL-17a, and tumor necrosis factor-α (Figures 4J–L). ADSC administration, however, made little impact on cytokines/chemokines or growth factor levels in serum (Figures 4A-L).

**Table 2:** Serum cytokine/ chemokine / growth factor analysis at 24 h after administration of MSCs.

		**Vehicle (*****n*** = **7)**		**ADSC (*****n*** = **8)**		**BM-MSC (*****n*** = **7)**		***P*** **value**	
		**Median**	**IQR**	**Median**	**IQR**	**Median**	**IQR**	**ADSC vs. vehicle**	**BM-MSC vs. vehicle**
Inflamatory cytokines	IL-1a	66.6	75.8	53.2	70.2	51.0	48.2	0.101	0.091
	IL-1b	89.6	18.7	78.7	42.1	77.9	56.9	0.288	0.2
	IL-5	244.6	61.8	255.5	49.1	298.9	68.2	0.953	0.146
	IL-6	580.1	465.1	473.4	327.6	804.4	453.2	0.666	0.999
	IL-17a	88.5	40.6	89.0	81.7	152.6	35.3	0.556	0.022
	IL-18	1882.0	4791.5	1603.5	1594.5	937.0	479.1	0.317	0.067
	TNFa	35.3	21.5	48.3	49.0	67.5	30.5	0.256	0.012
	INFγ	574.6	388.6	458.6	407.0	484.7	507.3	0.883	0.892
	CCL11 (Eotaxin)	52.2	17.3	48.0	17.0	58.0	13.8	0.805	0.67
	CXCL5 (LIX)	4695.0	1677.0	3473.5	1017.5	3025.0	1989.0	0.127	0.005
Anti-inflamatory cytokines	IL-2	115.5	67.8	147.6	137.6	212.4	65.9	0.274	0.016
	IL-4	87.2	49.5	106.9	82.8	148.4	46.6	0.742	0.068
	IL-12P70	657.1	370.4	785.3	849.7	1304.0	376.0	0.397	0.012
	IL-10	99.3	21.3	79.9	24.6	92.2	35.4	0.04	0.071
	IL-13	99.7	58.2	83.6	46.2	99.7	39.5	0.411	0.794
Chemotactic chemokines	CCL2 (MCP-1)	5947.0	1690.5	6023.5	2317.5	4878.0	1573.0	0.858	0.095
	CCL3 (MIP-1a)	78.4	31.3	73.1	17.3	47.6	17.3	0.606	0.009
	CCL5 (RANTES)	23243.0	18483.0	15889.5	12820.0	10978.0	3204.0	0.062	0.002
	CX3CL1 (Fractalkine)	208.2	61.9	186.5	106.0	153.0	9.8	0.188	0.012
	CXCL1 (GRO/KC)	245.7	102.8	158.9	164.6	116.8	60.6	0.125	0.017
	CXCL2 (MIP-2)	193.7	54.2	154.9	74.6	112.8	20.0	0.034	0.0001
	CXCL10 (IP-10)	650.0	472.7	376.7	233.1	421.6	170.1	0.003	0.006
Growth factors	GM-CSF	55.7	64.2	37.6	81.4	8.6	0.0	0.581	0.011
	G-CSF	30.3	23.7	36.3	46.7	65.8	33.1	0.838	0.045
	EGF	1.4	2.3	2.8	4.4	5.1	4.7	0.836	0.109
	VEGF	250.9	91.5	248.4	86.4	229.3	39.5	0.832	0.085

*IL, interleukin; TNF, tumor necrosis factor; INF, interferon; CCL, C-C motif chemokinne ligand; CXCL, C-X-C motif ligand; LIX, LPS-induced CXC chemokine; MCP, monocyte chemoattractant protein; MIP, macrophage inflammatory protein; RANTES, regulated on activation, normal T cell expressed and secreted; CX3CL, C-X3-C motif chemokine; IP, interferon gamma-induced protein; GM-CSF, granulocyte macrophage colony stimulating factor; G-CSF, granulocyte-colony stimulating factor; EGF, epidermal growth factor; VEGF, vascular endothelial growth factor*.

### Time-course of distribution and fate of ADSCS and BM-MSCS after intravenous injection

**Figure 5:**
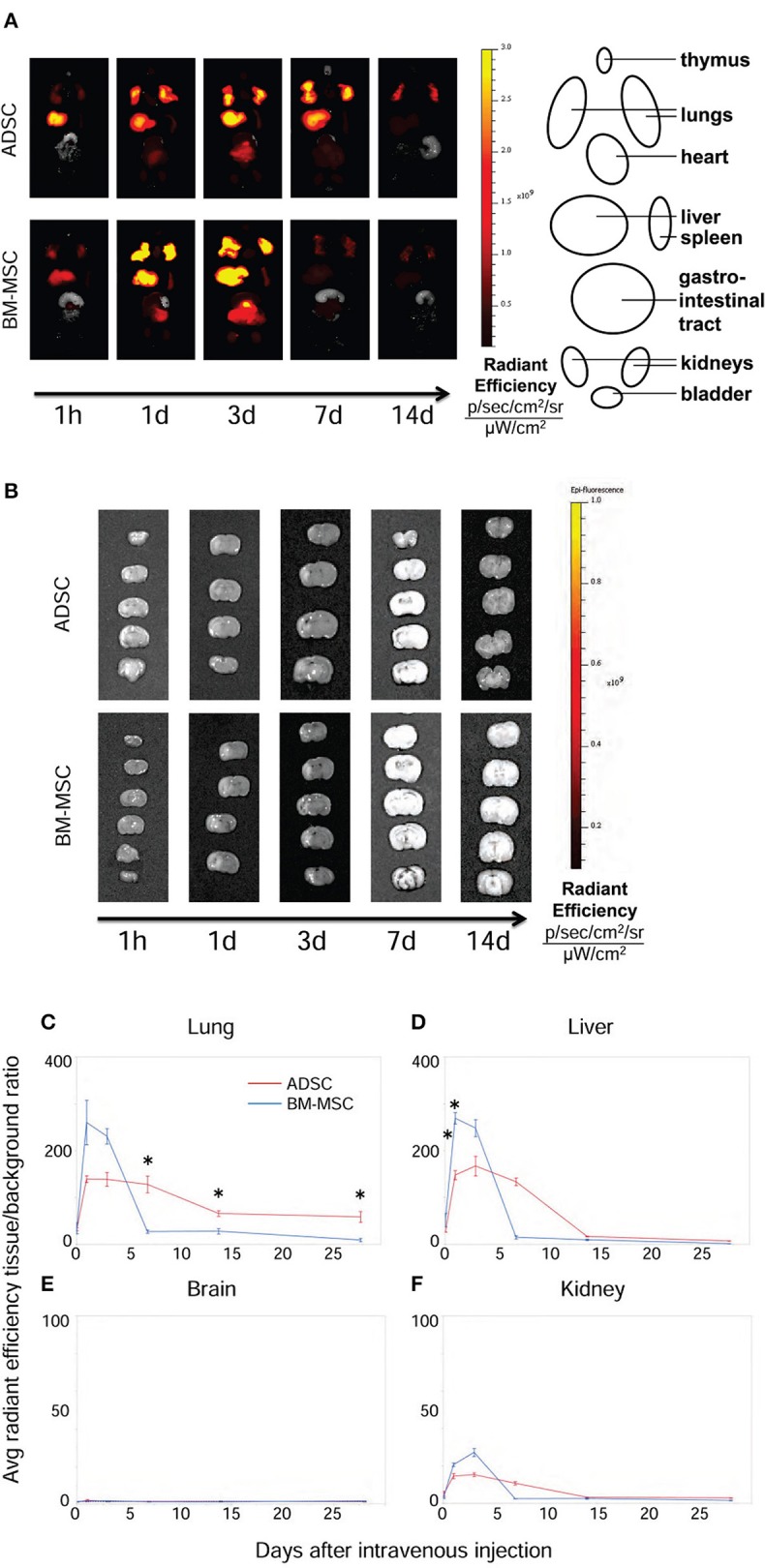
Distribution of ADSCs and BM-MSCs after intravenous injection. The distribution of ADSCs (*n* = 3) and BM-MSC (*n* = 3) after injection was detected by *ex vivo* imaging (IVIS®). Cells were labeled with DiR and injected 24 h after HI. ADSCs (red line) and BM-MSCs (blue line) mainly distributed into the lungs and liver in the first 3 d **(A,C,D)**. From 7 d after injection, the radiant efficiency of the lungs in ADSC-injected rats was significantly higher than those in the BM-MSC group **(A,C)**. Neither ADSCs nor BM-MSCs were detected in the brain at any time point **(B,E)**. There were no difference of fluorescence in the kidney **(F)**. *P < 0.05.

The distribution and fate of ADSCs and BM-MSCs after injection were evaluated serially by *ex vivo* imaging. Figure 5A shows representative pictures of the time-course of distribution in ADSC and BM-MSC groups. Both ADSCs and BM-MSCs mainly distributed in the lungs and liver within 3 d (Figures 5A,C,D). After that time, BM-MSC levels gradually reduced in the lungs. On the other hand, ADSCs remained in the lungs longer, even up to 28 d after injection (Figures 5A,C). No significant fluorescence was detected in the brain (Figures 5B,E) or kidney (Figure 5F) in either group at any time point.

### Pathological findings after injection of ADSCS or BM-MSCS 4 H after HI

**Figure 6:**
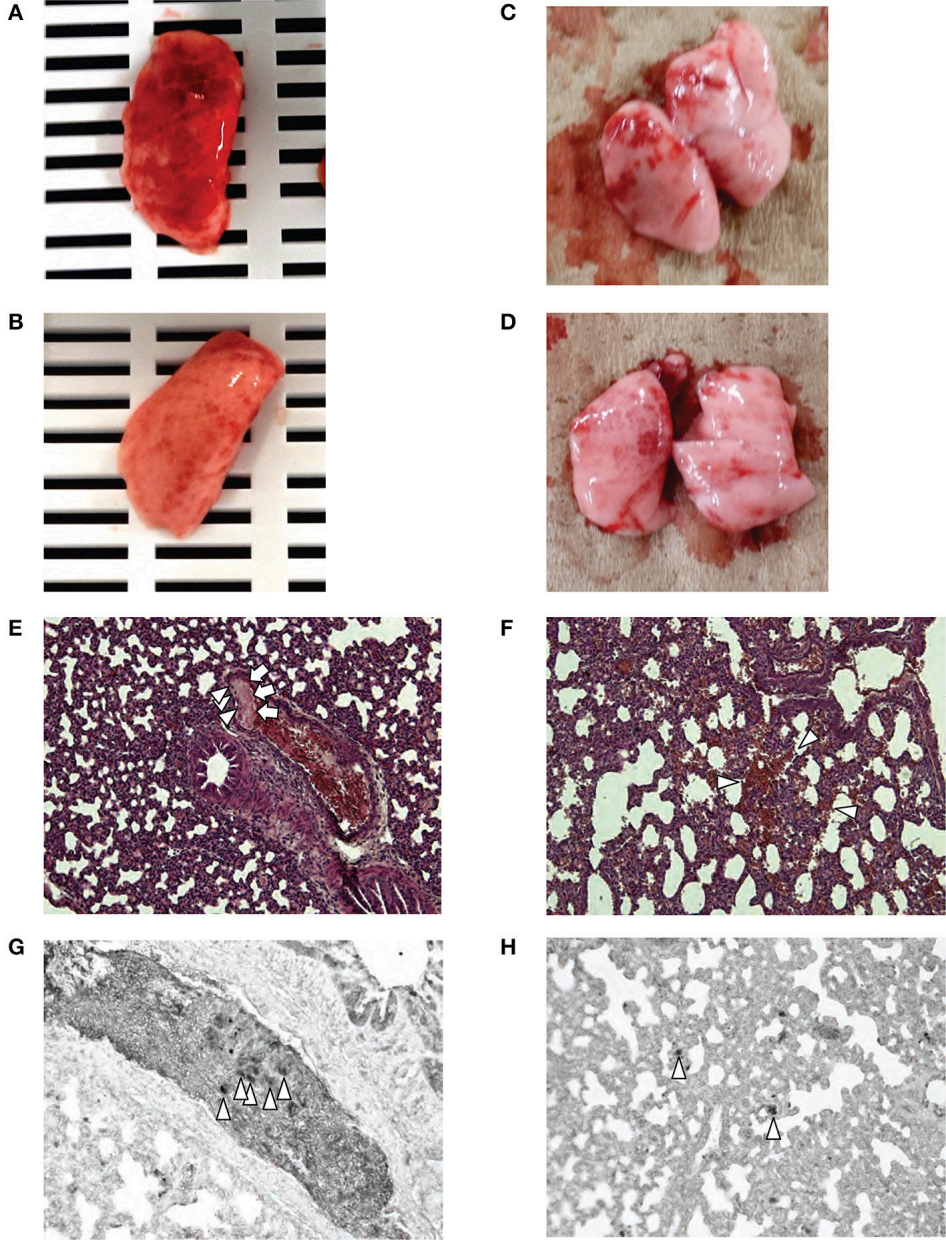
Pathological findings. Macroscopic evaluations after injection of 1 × 10^5^ ADSCs **(A)** and 1 × 10^4^ ADSCs **(B)** showed diffuse lung hemorrhages were markedly more severe in the ADSC group compared with rats injected with 1 × 10^5^ **(C)** and 1 × 10^4^ **(D)** BM-MSCs. Microscopic evaluation of the lungs after ADSC (1 × 10^6^ cells) injection [40 × magnification, HE] **(E)** revealed cell emboli (arrowhead) and fibrin deposition (arrow) in the large vessel. Fibrin deposition suggests embolism, inflammation, and coagulation. In the lungs of rats injected with 1 × 10^5^ ADSCs [200 × magnification, HE] **(F)**, diffuse alveolar hemorrhages were seen. Immunohistochemistry with anti-GFP **(G)** in the same blood vessel as **(E)** showed that cells with large nuclei filling pulmonary vessels were GFP-positive (arrowhead). The micrograph of lungs injected with 1 × 10^5^ ADSCs **(H)** showed the presence of many GFP-positive cells in the alveolar vessel (arrowhead).

Gross pathological observation after ADSC or BM-MSC injection revealed much more severe lung hemorrhaging in the ADSC group vs. the BM-MSC group (Figures 6A–D). The hemorrhages in the ADSC group were exacerbated as the number of cells injected increased. In rats given ADSC (1 × 10^5^ cells; *n* = 2), the hemorrhages were diffuse and filled the lung surface (Figure 6A). In rats given fewer ADSCs (1 × 10^4^ cells; *n* = 2), the hemorrhages were diffuse but not as extensively (Figure 6B). On the other hand, rats administered BM-MSCs (1 × 10^5^ or 1 × 10^4^ cells) showed sparse bleeding (Figures 6C,D).

Micropathologically, pulmonary embolism and alveolar hemorrhage were seen in both cell groups. Similarly, rats given ADSCs exhibited more severe pathology than BM-MSC rats, and severity increased by injected cell number. In the ADSC group (1 × 10^6^ cells; *n* = 2), pulmonary thrombosis by cells with large nuclei and fibrins were seen in some large vessels in the lung (Figure 6E) but not in the kidney or liver. This finding was compatible with pulmonary embolism. ADSC rats (1 × 10^5^ cells; *n* = 2) also had diffuse alveolar hemorrhages and fibrin deposition in small vessels; no special findings were seen in other organs. Administration of 1 × 10^5^ ADSCs per pup resulted in small vessel embolism and diffuse alveolar hemorrhage (Figure 6F). Immunohistopathology of lung tissue using anti-GFP showed that cells with large nuclei filling pulmonary vessels were GFP-positive (Figures 6G,H), which were injected cells derived from GFP-Tg rats. In contrast, BM-MSC rats (1 × 10^5^ or 1 × 10^6^ cells; *n* = 2) had cells with large nuclei in alveolar vessels, depending on the amount of injected cells, but hemorrhages and fibrin deposition were rare.

## Discussion

In the present study, we showed that intravenous administration of BM-MSCs had a therapeutic effect on HI brain injury in rats that resulted in reduction of apoptotic cells in the hippocampus. BM-MSC administration 24 h after HI decreased serum chemokine levels and increased anti-inflammatory cytokines. In addition, BM-MSCs also significantly decreased proinflammatory M1 microglia. In contrast, ADSC injection did not exhibit any such therapeutic effects but induced severe lung hemorrhaging and pulmonary embolism, leading to high mortality.

Herein, intravenous injection of BM-MSCs reduced apoptosis induced by HI in the CA3 area and dentate gyrus of the hippocampus, the most vulnerable areas to HI insult in premature brain (37). This finding corresponds with those we recently reported using mononuclear cells derived from human umbilical cord blood cells (7) and dedifferentiated fat cells (32). Furthermore, BM-MSCs have been shown to exert a therapeutic effect through different administration routes, including intracranial (38, 39), intracardiac (40), and intranasal (38).

One of the possible mechanisms related to this positive effect was that BM-MSC administration decreased M1 microglia. Recently, it was shown that not only macrophages but also microglia are divided into two types, proinflammatory (M1) and anti-inflammatory [M2] (41, 42). In the present study, BM-MSCs reduced the number of M1 microglia. This microglial change is one of the emerging targets for the treatment of neuronal injury or degenerative diseases (36, 43, 44). In the neonatal HI model, Donega et al. (45) also showed that intranasal administration of BM-MSCs decreased the M1 microglia, in accordance with our previous data showing the same change with intravenous injection of umbilical cord blood cells (8).

To elucidate further BM-MSC therapeutic mechanisms and support the observed microglial change, we also evaluated the impact of ADSC and BM-MSC administration on serum cytokine/chemokine levels in the current study. For the first time in neonatal HI rat models, we demonstrated serological amelioration of chemokines and anti-inflammatory cytokines by administration of BM-MSCs. Several proinflammatory chemokines/cytokines known to activate microglia were markedly decreased in the BM-MSC group, including CCL3, CX3CL1, CXCL1, CXCL2, CXCL3, and CXCL10 (46, 47). Thus, chemokine reduction, especially CX3CL1, is likely one mechanism by which BM-MSCs exert their therapeutic effect and reduce the M1 phenotype of microglia (48, 49). On the other hand, CCL2 levels, which are thought to decrease M1 phenotype, were decreased in our model. However, this chemokine is also known to activate circulating inflammatory monocytes (46). Therefore, its reduction may reflect an immunosuppressive effect of BM-MSC injection (50). Moreover, anti-inflammatory cytokines IL-2 and IL-4 were increased with BM-MSC injection. These anti-inflammatory cytokines are known to change microglial polarity M1 to M2 phenotype and are considered to be promising neuroprotective agents/targets (51). BM-MSC also significantly increased serum granulocyte colony stimulating factor levels. Granulocyte colony stimulating factor is also known as another agent that reduces M1 microglia (52). In adult stroke studies, many clinical trials using granulocyte colony stimulating factor are now undergoing (53). These chemokines/cytokines changes following BM-MSC administration support immunohistochemical findings on microglial status herein (i.e., reduced M1 phenotype).

Conversely, inflammatory cytokines, tumor necrosis factor-α and IL-12p70 were increased by BM-MSC injection. It has been shown that injection of MSCs alone can increase serum inflammatory cytokines levels (54). As the distribution of BM-MSCs was more systemic than that of ADSCs in the present study, BM-MSCs may be more likely to elevate reaction products in serum, whereas ADSCs remained in the lungs and induced local reactions.

The most amazing finding in the present study was that ADSC administration did not elicit any therapeutic effects (no change in apoptosis, microglial polarity, or serum chemokine/cytokine levels) but increased mortality instead. Pathologically, injection of 1 × 10^6^ ADSCs resulted in pulmonary embolisms with local inflammatory findings, and the embolisms contained many GFP-positive ADSCs. Even with a lower dose, (1 × 10^5^ cells) ADSC injection resulted in alveolar hemorrhage. There are some reports of MSCs having procoagulant activity when intravenously injected (29, 30, 55). However, it is unknown why ADSCs are more likely to cause such a response. Shiratsuki et al. (30) reported that ADSCs, but not BM-MSCs markedly increased prothrombin time, indicating that ADSCs potentially enhance procoagulation activity. In addition, we showed that ADSCs remained in the lungs longer than BM-MSCs after injection. A longer stay in the lungs can exacerbate the negative features of ADSCs action. In the present study, severe pulmonary embolisms were seen but were not present in the kidneys. If the procoagulant effect of ADSCs is systemic, like disseminated intravascular coagulation (DIC), similar findings should also be seen in the kidneys. Therefore, the hypercoagulable condition is thought to be due to a local reaction. The longer time spent by ADSCs in the lung may partially explain the local reactions observed therein.

One possible reason why ADSCs remain longer in the lungs than BM-MSCs may be due to different expression of cell adhesion molecules. Yang et al. (56) reported that BM-MSCs express CD106 (vascular cell adhesion molecule-1), while ADSCs do not. Vascular cell adhesion molecule-1 plays an important role in cellular adhesion to vessels (57). Intravenously injected cells first encounter blood capillaries in the lungs. Therefore, the lack of such an adhesion molecule may cause a “rough landing” on the capillaries, resulting in inflammation and embolism. Moreover, another previous report revealed that allogenic BM-MSCs upregulate urokinase plasminogen activator expression in a mouse model of pulmonary embolism (58). Thus, BM-MSCs may inherently prevent emboli.

There are two limitations in the present study. One limitation is that there is no evaluation of M2 microglia. Considering the cytokine/chemokine result in the present study and previous publications (8, 45), changing microglial polarity from M1 into M2 is most plausible, but it is not shown in the present study. The other limitation is that each cell type was injected intravenously using simple preparations (i.e., suspended in 0.1 mL of PBS without any measures). A safe method of ADSC injection has not yet been fully investigated, but there are reports on countermeasures against embolism. For example, Yukawa et al. (59) showed that co-administration of an antithrombin agent prevented lung entrapment of ADSCs in a rodent model. As another countermeasure, cell culture methods may be able to improve development of embolisms. Our low serum-cultured ADSCs (23) have been shown to effectively ameliorate kidney disease in a rodent model via intravenous administration (20). In our preliminary experiments, low serum-cultured ADSCs could be administered to the present HI model as safely as BM-MSCs without adding antithrombin agent (Supplemental Table).

## Conclusion

Intravenous injection of allogeneic BM-MSCs, but not ADSCs, ameliorated neonatal rat HI injury by reducing M1 microglia and suppressing expression of inflammatory cytokines/chemokines. In contrast, administration of ADSCs induced severe lung hemorrhage and higher mortality.

## Author contributions

YuS, YK, TS, TK, and AM were actively involved in animal experiments. SS supported cell preparation. YuS, YSat, SM, and TY conceptualized and designed the study. YuS, YSat, AH, SM, MT, TY, and MH interpreted the data. YShi performed pathological evaluation. YuS drafted the initial manuscript, and YSat, MT, and MH critically reviewed the manuscript. All authors approved the final manuscript as submitted and agree to be accountable for all aspects of the work.

### Conflict of interest statement

The authors declare that the research was conducted in the absence of any commercial or financial relationships that could be construed as a potential conflict of interest.

## Abbreviations

ADSC: adipose tissue-derived stem cells
BM-MSC: bone marrow-derived mesenchymal stem cells
CA: cornu ammonis
DAPI: 4',6-diamidino-2-phenylindole
DiR: 1,1-dioctadecyl-3,3,3,3-tetramethyl indotricarbocyanine iodide
EDTA: ethylenediaminetetraacetic acid
FBS: fetal bovine serum
GFP: green fluorescent protein
Tg: transgenic
HI: hypoxic-ischemic
Iba1: ionized calcium-binding adapter molecule 1
iNOS: inducible nitric oxide synthase
MEM: modified Eagle's medium
P7: postnatal day 7
PBS: phosphate-buffered saline
DIC: disseminated intravascular coagulation.

## References

[1] Kurinczuk JJ White-Koning M Badawi N. Epidemiology of neonatal encephalopathy and hypoxic-ischaemic encephalopathy. Early Hum Dev. (2010) 86:329–38. 10.1016/j.earlhumdev.2010.05.010 20554402

[2] Hayakawa M Ito Y Saito S Mitsuda N Hosono S Yoda H. Incidence and prediction of outcome in hypoxic-ischemic encephalopathy in Japan. Pediatr Int. (2014) 56:215–21. 10.1111/ped.12233 24127879 PMC4491348

[3] Gluckman PD Wyatt JS Azzopardi D Ballard R Edwards AD Ferriero DM. Selective head cooling with mild systemic hypothermia after neonatal encephalopathy: multicentre randomised trial. Lancet (2005) 365:663–70. 10.1016/S0140-6736(05)17946-X 15721471

[4] Shah PS Ohlsson A Perlman M. Hypothermia to treat neonatal hypoxic ischemic encephalopathy: systematic review. Arch Pediatr Adolesc Med. (2007) 161:951–8. 10.1001/archpedi.161.10.951 17909138

[5] Shankaran S Pappas A Mcdonald SA Vohr BR Hintz SR Yolton K. Childhood outcomes after hypothermia for neonatal encephalopathy. N Engl J Med. (2012) 366:2085–92. 10.1056/NEJMoa1112066 22646631 PMC3459579

[6] Tsuji M Taguchi A Ohshima M Kasahara Y Sato Y Tsuda H. Effects of intravenous administration of umbilical cord blood CD34(+) cells in a mouse model of neonatal stroke. Neuroscience (2014) 263:148–58. 10.1016/j.neuroscience.2014.01.018 24444827

[7] Hattori T Sato Y Kondo T Ichinohashi Y Sugiyama Y Yamamoto M. Administration of umbilical cord blood cells transiently decreased hypoxic-ischemic brain injury in neonatal rats. Dev Neurosci. (2015) 37:95–104. 10.1159/000368396 25720519

[8] Nakanishi K Sato Y Mizutani Y Ito M Hirakawa A Higashi Y. Rat umbilical cord blood cells attenuate hypoxic-ischemic brain injury in neonatal rats. Sci Rep. (2017) 7:44111. 10.1038/srep44111 28281676 PMC5345001

[9] Cotten CM Murtha AP Goldberg RN Grotegut CA Smith PB Goldstein RF. Feasibility of autologous cord blood cells for infants with hypoxic-ischemic encephalopathy. J Pediatr. (2014) 164:973–9.e71. 10.1016/j.jpeds.2013.11.036 24388332 PMC3992180

[10] Sato Y Nakanishi K Hayakawa M Kakizawa H Saito A Kuroda Y. Reduction of brain injury in neonatal hypoxic-ischemic rats by intracerebroventricular injection of neural stem/progenitor cells together with chondroitinase ABC. Reprod Sci. (2008) 15:613–20. 10.1177/1933719108317299 18579850

[11] Sato Y Oohira A. Chondroitin sulfate, a major niche substance of neural stem cells, and cell transplantation therapy of neurodegeneration combined with niche modification. Curr Stem Cell Res Ther. (2009) 4:200–9. 10.2174/157488809789057419 19492981

[12] Gonzalez MA Gonzalez-Rey E Rico L Buscher D Delgado M. Adipose-derived mesenchymal stem cells alleviate experimental colitis by inhibiting inflammatory and autoimmune responses. Gastroenterology (2009) 136:978–89. 10.1053/j.gastro.2008.11.041 19135996

[13] Nakao N Nakayama T Yahata T Muguruma Y Saito S Miyata Y. Adipose tissue-derived mesenchymal stem cells facilitate hematopoiesis *in vitro* and *in vivo*: advantages over bone marrow-derived mesenchymal stem cells. Am J Pathol. (2010) 177:547–54. 10.2353/ajpath.2010.091042 20558580 PMC2913350

[14] Van Velthoven CT Kavelaars A Van Bel F Heijnen CJ. Mesenchymal stem cell treatment after neonatal hypoxic-ischemic brain injury improves behavioral outcome and induces neuronal and oligodendrocyte regeneration. Brain Behav Immun. (2010) 24:387–93. 10.1016/j.bbi.2009.10.017 19883750

[15] Chen J Tang YX Liu YM Chen J Hu XQ Liu N. Transplantation of adipose-derived stem cells is associated with neural differentiation and functional improvement in a rat model of intracerebral hemorrhage. CNS Neurosci Ther. (2012) 18:847–54. 10.1111/j.1755-5949.2012.00382.x 22934896 PMC6493568

[16] Van Velthoven CT Kavelaars A Heijnen CJ. Mesenchymal stem cells as a treatment for neonatal ischemic brain damage. Pediatr Res. (2012) 71:474–81. 10.1038/pr.2011.64 22430383

[17] Yang KL Lee JT Pang CY Lee TY Chen SP Liew HK. Human adipose-derived stem cells for the treatment of intracerebral hemorrhage in rats via femoral intravenous injection. Cell Mol Biol Lett. (2012) 17:376–92. 10.2478/s11658-012-0016-5 22544763 PMC6275678

[18] Schaffler A Buchler C. Concise review: adipose tissue-derived stromal cells–basic and clinical implications for novel cell-based therapies. Stem Cells (2007) 25:818–27. 10.1634/stemcells.2006-0589 17420225

[19] Wei X Du Z Zhao L Feng D Wei G He Y. IFATS collection: the conditioned media of adipose stromal cells protect against hypoxia-ischemia-induced brain damage in neonatal rats. Stem Cells (2009) 27:478–88. 10.1634/stemcells.2008-0333 19023032

[20] Katsuno T Ozaki T Saka Y Furuhashi K Kim H Yasuda K. Low serum cultured adipose tissue-derived stromal cells ameliorate acute kidney injury in rats. Cell Transplant. (2013) 22:287–97. 10.3727/096368912X655019 22963874

[21] Chan TM Chen JY Ho LI Lin HP Hsueh KW Liu DD. ADSC therapy in neurodegenerative disorders. Cell Transplant. (2014) 23:549–57. 10.3727/096368914X678445 24816450

[22] Frazier TP Mclachlan JB Gimble JM Tucker HA Rowan BG. Human adipose-derived stromal/stem cells induce functional CD4+CD25+FoxP3+CD127- regulatory T cells under low oxygen culture conditions. Stem Cells Dev. (2014) 23:968–77. 10.1089/scd.2013.0152 24405386 PMC3997145

[23] Iwashima S Ozaki T Maruyama S Saka Y Kobori M Omae K. Novel culture system of mesenchymal stromal cells from human subcutaneous adipose tissue. Stem Cells Dev. (2009) 18:533–43. 10.1089/scd.2008.0358 19055360

[24] Zuk PA Zhu M Mizuno H Huang J Futrell JW Katz AJ. Multilineage cells from human adipose tissue: implications for cell-based therapies. Tissue Eng. (2001) 7:211–28. 10.1089/107632701300062859 11304456

[25] Ikegame Y Yamashita K Hayashi S Mizuno H Tawada M You F. Comparison of mesenchymal stem cells from adipose tissue and bone marrow for ischemic stroke therapy. Cytotherapy (2011) 13:675–85. 10.3109/14653249.2010.549122 21231804

[26] Frese L Dijkman PE Hoerstrup SP. Adipose tissue-derived stem cells in regenerative medicine. Transfus Med Hemother. (2016) 43:268–74. 10.1159/000448180 27721702 PMC5040903

[27] Toyserkani NM Jorgensen MG Tabatabaeifar S Jensen CH Sheikh SP Sorensen JA. Concise review: a safety assessment of adipose-derived cell therapy in clinical trials: a systematic review of reported adverse events. Stem Cells Transl Med. (2017) 6:1786–94. 10.1002/sctm.17-0031 28722289 PMC5689766

[28] Boltze J Arnold A Walczak P Jolkkonen J Cui L Wagner DC. The dark side of the force - constraints and complications of cell therapies for stroke. Front Neurol. (2015) 6:155. 10.3389/fneur.2015.00155 26257702 PMC4507146

[29] Tatsumi K Ohashi K Matsubara Y Kohori A Ohno T Kakidachi H. Tissue factor triggers procoagulation in transplanted mesenchymal stem cells leading to thromboembolism. Biochem Biophys Res Commun. (2013) 431:203–9. 10.1016/j.bbrc.2012.12.134 23313481

[30] Shiratsuki S Terai S Murata Y Takami T Yamamoto N Fujisawa K. Enhanced survival of mice infused with bone marrow-derived as compared with adipose-derived mesenchymal stem cells. Hepatol Res. (2015) 45:1353–9. 10.1111/hepr.12507 25692387

[31] Rice JRIII Vannucci RC Brierley JB. The influence of immaturity on hypoxic-ischemic brain damage in the rat. Ann Neurol. (1981) 9:131–41. 10.1002/ana.410090206 7235629

[32] Mikrogeorgiou A Sato Y Kondo T Hattori T Sugiyama Y Ito M. Dedifferentiated fat cells as a novel source for cell therapy to target neonatal hypoxic-ischemic encephalopathy. Dev Neurosci. (2017) 39:273–86. 10.1159/000455836 28273662

[33] Cho H Indig GL Weichert J Shin HC Kwon GS. *In vivo* cancer imaging by poly(ethylene glycol)-b-poly(varepsilon-caprolactone) micelles containing a near-infrared probe. Nanomedicine (2012) 8:228–36. 10.1016/j.nano.2011.06.009 21704593 PMC3193583

[34] Ruan J Song H Li C Bao C Fu H Wang K. DiR-labeled embryonic stem cells for targeted imaging of *in vivo* gastric cancer cells. Theranostics (2012) 2:618–28. 10.7150/thno.4561 22768029 PMC3388594

[35] Osato K Sato Y Ochiishi T Osato A Zhu C Sato M. Apoptosis-inducing factor deficiency decreases the proliferation rate and protects the subventricular zone against ionizing radiation. Cell Death Dis. (2010) 1:e84. 10.1038/cddis.2010.63 21368857 PMC3035904

[36] Matsubara K Matsushita Y Sakai K Kano F Kondo M Noda M. Secreted ectodomain of sialic acid-binding Ig-like lectin-9 and monocyte chemoattractant protein-1 promote recovery after rat spinal cord injury by altering macrophage polarity. J Neurosci. (2015) 35:2452–64. 10.1523/JNEUROSCI.4088-14.2015 25673840 PMC6605605

[37] George SA Barrett RD Bennet L Mathai S Jensen EC Gunn AJ. Nonadditive neuroprotection with early glutamate receptor blockade and delayed hypothermia after asphyxia in preterm fetal sheep. Stroke (2012) 43:3114–7. 10.1161/STROKEAHA.112.671982 22923445

[38] Van Velthoven CT Kavelaars A Van Bel F Heijnen CJ. Nasal administration of stem cells: a promising novel route to treat neonatal ischemic brain damage. Pediatr Res. (2010) 68:419–22. 10.1203/PDR.0b013e3181f1c289 20639794

[39] Gu Y Zhang Y Bi Y Liu J Tan B Gong M. Mesenchymal stem cells suppress neuronal apoptosis and decrease IL-10 release via the TLR2/NFkappaB pathway in rats with hypoxic-ischemic brain damage. Mol Brain (2015) 8:65. 10.1186/s13041-015-0157-3 26475712 PMC4609057

[40] Lee JA Kim BI Jo CH Choi CW Kim EK Kim HS. Mesenchymal stem-cell transplantation for hypoxic-ischemic brain injury in neonatal rat model. Pediatr Res. (2010) 67:42–6. 10.1203/PDR.0b013e3181bf594b 19745781

[41] Taylor RA Sansing LH. Microglial responses after ischemic stroke and intracerebral hemorrhage. Clin Dev Immunol. (2013) 2013:746068. 10.1155/2013/746068 24223607 PMC3810327

[42] Franco R Fernandez-Suarez D. Alternatively activated microglia and macrophages in the central nervous system. Prog Neurobiol. (2015) 131:65–86. 10.1016/j.pneurobio.2015.05.003 26067058

[43] Zanier ER Pischiutta F Riganti L Marchesi F Turola E Fumagalli S. Bone marrow mesenchymal stromal cells drive protective M2 microglia polarization after brain trauma. Neurotherapeutics (2014) 11:679–95. 10.1007/s13311-014-0277-y 24965140 PMC4121458

[44] Park HJ Oh SH Kim HN Jung YJ Lee PH. Mesenchymal stem cells enhance alpha-synuclein clearance via M2 microglia polarization in experimental and human parkinsonian disorder. Acta Neuropathol. (2016) 132:685–701. 10.1007/s00401-016-1605-6 27497943

[45] Donega V Nijboer CH Van Tilborg G Dijkhuizen RM Kavelaars A Heijnen CJ. Intranasally administered mesenchymal stem cells promote a regenerative niche for repair of neonatal ischemic brain injury. Exp Neurol. (2014) 261:53–64. 10.1016/j.expneurol.2014.06.009 24945601

[46] Benakis C Garcia-Bonilla L Iadecola C Anrather J. The role of microglia and myeloid immune cells in acute cerebral ischemia. Front Cell Neurosci. (2014) 8:461. 10.3389/fncel.2014.00461 25642168 PMC4294142

[47] Mayer AM Murphy J Macadam D Osterbauer C Baseer I Hall ML. Classical and Alternative activation of *Cyanobacterium oscillatoria* sp. Lipopolysaccharide-treated rat microglia in vitro. Toxicol Sci. (2016) 149:484–95. 10.1093/toxsci/kfv251 26609141 PMC4900220

[48] Fumagalli S Perego C Ortolano F De Simoni MG. CX3CR1 deficiency induces an early protective inflammatory environment in ischemic mice. Glia (2013) 61:827–42. 10.1002/glia.22474 23440897

[49] Tang Z Gan Y Liu Q Yin JX Liu Q Shi J. CX3CR1 deficiency suppresses activation and neurotoxicity of microglia/macrophage in experimental ischemic stroke. J Neuroinflammat. (2014) 11:26. 10.1186/1742-2094-11-26 24490760 PMC3942808

[50] Cagliani J Grande D Molmenti EP Miller EJ Rilo HLR. Immunomodulation by mesenchymal stromal cells and their clinical applications. J Stem Cell Regen Biol. (2017) 3. 10.15436/2471-0598.17.022 29104965 PMC5667922

[51] Cherry JD Olschowka JA O'banion MK. Neuroinflammation and M2 microglia: the good, the bad, and the inflamed. J Neuroinflam. (2014) 11:98. 10.1186/1742-2094-11-98 24889886 PMC4060849

[52] Guo Y Zhang H Yang J Liu S Bing L Gao J. Granulocyte colony-stimulating factor improves alternative activation of microglia under microenvironment of spinal cord injury. Neuroscience (2013) 238:1–10. 10.1016/j.neuroscience.2013.01.047 23419550

[53] Huang X Liu Y Bai S Peng L Zhang B Lu H. Granulocyte colony stimulating factor therapy for stroke: a pairwise meta-analysis of randomized controlled trial. PLoS ONE (2017) 12:e0175774. 10.1371/journal.pone.0175774 28406964 PMC5391086

[54] Hoogduijn MJ Roemeling-Van Rhijn M Engela AU Korevaar SS Mensah FK Franquesa M. Mesenchymal stem cells induce an inflammatory response after intravenous infusion. Stem Cells Dev. (2013) 22:2825–35. 10.1089/scd.2013.0193 23767885

[55] Gleeson BM Martin K Ali MT Kumar AH Pillai MG Kumar SP. Bone marrow-derived mesenchymal stem cells have innate procoagulant activity and cause microvascular obstruction following intracoronary delivery: amelioration by antithrombin therapy. Stem Cells (2015) 33:2726–37. 10.1002/stem.2050 25969127

[56] Yang ZX Han ZB Ji YR Wang YW Liang L Chi Y. CD106 identifies a subpopulation of mesenchymal stem cells with unique immunomodulatory properties. PLoS ONE (2013) 8:e59354. 10.1371/journal.pone.0059354 23555021 PMC3595282

[57] Elices MJ Osborn L Takada Y Crouse C Luhowskyj S Hemler ME. VCAM-1 on activated endothelium interacts with the leukocyte integrin VLA-4 at a site distinct from the VLA-4/fibronectin binding site. Cell (1990) 60:577–84. 10.1016/0092-8674(90)90661-W 1689216

[58] Peng X Li J Yu X Tan R Zhu L Wang J. Therapeutic effectiveness of bone marrow-derived mesenchymal stem cell administration against acute pulmonary thromboembolism in a mouse model. Thromb Res. (2015) 135:990–9. 10.1016/j.thromres.2015.02.009 25712897

[59] Yukawa H Watanabe M Kaji N Okamoto Y Tokeshi M Miyamoto Y. Monitoring transplanted adipose tissue-derived stem cells combined with heparin in the liver by fluorescence imaging using quantum dots. Biomaterials (2012) 33:2177–86. 10.1016/j.biomaterials.2011.12.009 22192539

